# Salivary gland maturation and duct formation in the African malaria mosquito *Anopheles gambiae*

**DOI:** 10.1038/s41598-017-00672-0

**Published:** 2017-04-04

**Authors:** Michael B. Wells, Jordan Villamor, Deborah J. Andrew

**Affiliations:** 1 Department of Cell Biology, The Johns Hopkins University School of Medicine, 725N. Wolfe St., Baltimore, MD 21205 USA; 2 The Johns Hopkins Malaria Research Institute, The Johns Hopkins University School of Medicine, 725N. Wolfe St., Baltimore, MD 21205 USA

## Abstract

Mosquito-borne diseases cause one million deaths and hundreds of millions of human infections yearly. With all such diseases, the pathogen must traverse the mosquito salivary gland (SG) for transmission to a new host, making the SGs ideal targets for genetic strategies to block transmission. Prior studies have elucidated details of SG structure by light and electron microscopy and have deeply explored the salivary transcriptome and proteome. Very little is known, however, about how the unique functional architecture of mosquito SGs is achieved. Using immunohistochemistry and confocal microscopy, we address two questions regarding SGs of the malaria vector *Anopheles gambiae*. How does the distinct cup-shaped morphology of SG secretory cells arise? And, how does the salivary duct, the structure through which saliva and parasites exit the glands, form? We demonstrate that SG cells begin as cuboidal-shaped cells surrounding a matrix-filled lumen that mature into cup-shaped cells through the formation and fusion of a large pre-apical compartment (PAC) to the apical surface. The secretory duct begins as buds of chitin at the apical surface of individual secretory cells. Further chitin deposition connects these chitin buds to form a contiguous duct that largely separates from the apical surface during PAC fusion.

## Introduction

Mosquitoes represent a major threat to human health. Mosquito-borne disease accounts for nearly one million deaths and hundreds of millions of infections annually. Mosquito control efforts in areas hardest hit by mosquito-borne diseases, such as Africa, focus on insecticide-treated bed nets and insecticide spray treatments. These interventions have markedly reduced deaths since the year 2000, but resistance to insecticides continues to grow, and insecticides may not prevent neighboring vector species from invading cleared territory. Recent advances have focused on disrupting mosquito reproduction using selfish genes as one disease containment strategy^1, 2^, an approach likely to elicit significant selection pressures. Since nearly all mosquito-borne diseases require the pathogen to traverse the mosquito salivary glands for transmission^3^, the salivary glands (SGs) are an excellent organ to target using gene drive strategies. Indeed, recent studies showed that using a mouse cell death activator gene to compromise SG survival in mosquitoes not only limited parasite transmission, but had little measurable effect on organismal viability or fecundity^4^.

Despite its importance in the life cycle of insect-borne pathogens, very little is known about molecular and cellular events underlying the specification, maturation or maintenance of the adult salivary gland in any insect. Formation of the adult mosquito SGs begins during pupal development^5–7^; time to maturation varies from three to seven days post eclosion, depending on the species^8–10^. Electron microscopy data from limited samples, as well as somewhat broader studies using immunohistochemistry and light microscopy, have shown that mature adult mosquito SG lobes are composed of cells that are generally cup shaped, encasing a large apical secretory cavity connected to a salivary duct and/or lumen^5, 9–15^. Variation in SG lobe number has been observed multiple times^5, 16, 17^. The large secretory cavity of SG cells is known to function as a holding tank for infective parasites^3, 18^. The salivary duct, which is often only slightly wider than a *Plasmodium* parasite^11^, is the pathogens’ escape route from the mosquito to a new host^13^. Until now, the cellular mechanisms of how the unusual cup-shaped morphology of secretory cells is achieved and the cellular origin of the salivary duct were unknown. Here, we show that the cup-shaped secretory structure evolves from a more simple cuboidal morphology and that the secretory portion of the duct comes from the secretory cells.

## Results and Discussion

### Early adult SG cells are cuboidal and produce apical WGA-positive chitin

**Figure 1:**
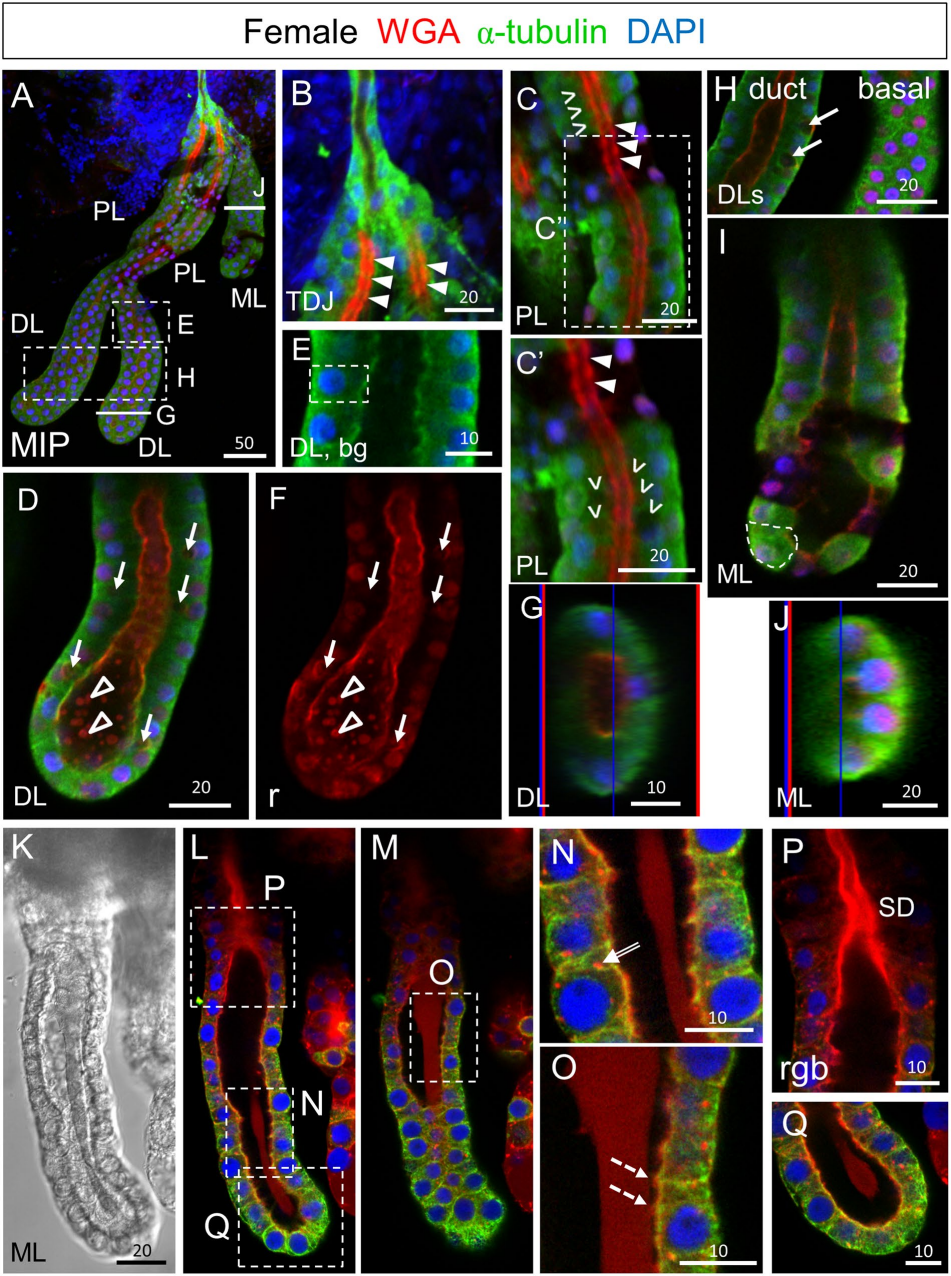
SGs from newly eclosed adult females comprise cuboidal secretory cells and a central WGA-positive maturing apical duct. (**A**) Low magnification maximum intensity “Z” projection (MIP) confocal image of a SG about 30 minutes post eclosion stained with antibodies to newly synthesized alpha-tubulin (green; tubulin antibody) and the dyes WGA (red; O-GlcNAcylated proteins and chitin) and DAPI (blue; nuclei). (**B**) Confocal image of the proximal triductal junction (TDJ) region showing high levels of alpha-tubulin in the cell bodies of duct cells and high levels of periodic WGA staining in the apical duct region of the most proximal secretory cells. Arrowheads indicate lower intensity staining near cell junctions. (**C**) Confocal image of the proximal lobe (PL) with variable levels of alpha-tubulin staining of cuboidal-shaped cells and high-level periodic apical WGA staining in the duct region. Arrowheads indicate lower intensity staining near cell junctions. Carets mark sites of secretory cavity formation. (**C’**) Zoom image of (**C**). (**D**–**F**) Single confocal images of the distal lobe (DL) showing cuboidal shaped cells (dashed outline, **E**) with high levels of WGA staining at the apical surface as well as lower and particulate WGA staining in the lumen (open arrowheads). Arrows (**D**,**F**) indicate pre-apical compartments (PACs). (**G**) Cross section of the DL reveals cuboidal alpha-tubulin positive secretory cells with apical WGA staining surrounding the central lumen. The thin blue and red lines (**G**,**J**) are positional markers added by the “Ortho” function in the Zen software. (**H**) Confocal images of DLs showing a central (duct) section (left) or a basal section (right). Arrows indicate PACs. (**I**,**J**) Sagittal and cross sections of the medial lobe with variable staining of alpha-tubulin and low level apical WGA staining. The dashed line (**I**) highlights the cuboidal shape of the cells. (**K**–**Q**) Early ML showing faint WGA staining of the apical matrix. (**K**) DIC image of ML. (**L**,**M**) Different focal planes of same gland showing the apical matrix either pulled away from or contacting the cuboidal secretory cells. (**N**) WGA-positive vesicles are observed within secretory cells of the ML (double-lined arrow). (**O**) The apical matrix directly contacts the apical domains of the secretory cells in some areas (dashed arrows). (**P**,**Q**) Zoomed images of (**L**). Individual channels are denoted “b”–blue, DAPI, “g”–green, tubulin, or “r”–red, WGA. Scale bar lengths are in microns. No primary antibody control imaging is shown in Supplementary Figure S1.

To learn how the unusual morphology of the SG cell arises and to characterize morphological variation in the African malaria vector *Anopheles gambiae*, we applied an optimized method for dye and immunohistochemical (IHC) staining to SGs isolated from newly eclosed adults and from adults several days post eclosion^19^. We began by interrogating SGs from adults 30 minutes post eclosion using an alpha-tubulin antibody (AA4.3), DAPI (DNA), and Rhodamine-conjugated Wheat Germ Agglutinin (Rh-WGA), a lectin dye that binds chitin and O-GlcNAcylated proteins (Fig. 1). Female SGs typically had two elongated lateral lobes and a single medial lobe (ML), which met at the triductal junction (TDJ) to connect to the more proximal duct cells. Duct cells at the TDJ had high levels of AA4.3 alpha-tubulin staining and undetectable levels of apical WGA staining (Fig. 1A,B). At this stage, cells of the proximal lateral lobe (PL), which showed highly variable alpha-tubulin staining, were primarily cuboidal in shape, with a subset showing small apical secretory cavities (areas that lack alpha-tubulin staining in the alpha-tubulin-positive cell bodies; Fig. 1C–C’, carets) adjacent to the apical surface, which had high levels of WGA staining, likely corresponding to newly synthesized chitin (Fig. 1B–C’). WGA staining in the PL was periodic with high-level staining extending across the apical cell surfaces interrupted by low-level staining at cell boundaries (Fig. 1B–C’, arrowheads). AA4.3 alpha-tubulin staining also varied within both PL and DL cells, often showing higher intensity near the basal and sometimes apical surfaces (1C, E, H). This increased alpha-tubulin intensity within different cellular domains could function to stabilize cell structure.

In SGs of newly eclosed adults, almost all distal lateral lobe (DL) cells were cuboidal (Fig. 1D–H) and many had large round internal compartments that did not stain for alpha-tubulin and frequently exhibited peripheral WGA staining; we refer to these as pre-apical compartments [(PACs); Fig. 1D,F, arrows]. High WGA staining was also observed along the apical surfaces of cells throughout the DL, with additional staining of punctate lumenal structures at the most distal end of the tube (Fig. 1D,F, open arrowheads). A cross-section through the DL revealed that the cell bodies directly contacted the WGA positive chitin-lined lumen (Fig. 1G). At this stage, all MLs contained cuboidal cells with round nuclei and variable apical and lumenal WGA staining (Fig. 1I–Q). In some MLs, detectable WGA staining was limited to the apical surfaces (Fig. 1I), whereas others showed additional low-level uniform WGA staining in the lumen directly contacting the apical surface of ML cells in some regions (Fig. 1O, dashed arrows). Punctate cellular staining was also often observed (Fig. 1N, double arrow). Apical WGA staining was almost always higher in the most proximal region of the ML (Fig. 1L,P). In summary, newly eclosed adult SG cells are mostly cuboidal, not cup-shaped. They produce and secrete apical chitin that is likely to contribute to the developing secretory duct. The cuboidal cells of the PL and DL also contained subcellular PACs, the large round internal structure lacking tubulin signal.

### Formation of PL and DL cup-shaped cells occurs within the next ~24 hours

**Figure 2:**
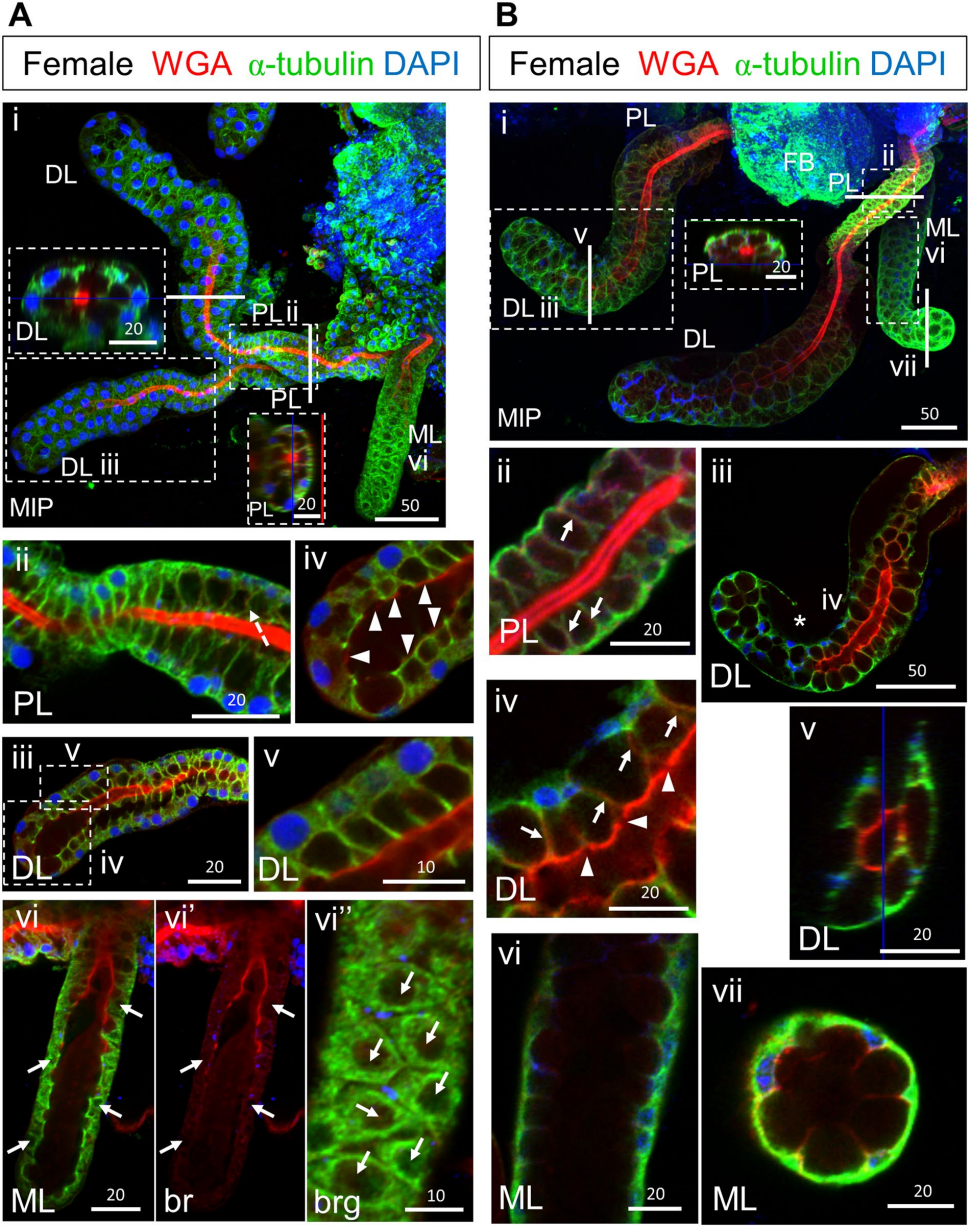
Female SG cells undergo an architectural transition to attain their final cup shape. Low magnification maximum intensity “Z” projection (MIP) confocal images of female SGs either (Ai) 12–36 hours post eclosion or (Bi) four days post eclosion stained with antibodies to newly synthesized alpha-tubulin (green) and the dyes WGA (red) and DAPI (blue). (Ai) By mid/late day one post eclosion, SG secretory cavities (SCs) are evident (Ai, insets). The thin red and blue lines (Ai inset, Bi inset, Bv) are positional markers added by the “Ortho” function in the Zen software. (Aii) In the PL, individual cells are cup-shaped with secretory cavities (SCs) of variable size. In many cells, the cell body at the base of the “cups” remains thick, and the nuclei are mostly round. The walls of the cup are sometimes bent (dashed arrow). (Aiii,iv) Similarly, DL cells are cup-shaped, with only moderately compressed cell bodies. The rims of the cup-shaped cells directly contact the WGA-positive secretory duct in all but the most distal DL cells where some cells also appear to have recently fused PACs. Arrowheads indicate apical WGA staining. Some cells were severed in Aiii during IHC processing. (Avi–vi″) Cells in the ML appear compressed with only slight indentations on the surface where the secretory cavities will eventually form. (Avi) Nuclei are smaller and are primarily located at the basal periphery. A confocal slice through the cell bodies reveals PACs (nearly round compartments depleted of alpha-tubulin staining) in nearly all cells (Avi–Avi”, arrows). (Bi) By day four post eclosion, SCs have completed maturation and expansion. In all lobes, SCs occupy a much larger volume than the surrounding cup-shaped cell bodies, WGA and alpha-tubulin are observed in cells, and nuclei are basally positioned. (Bii) A dense WGA stained duct is present in the PL. WGA signal is also seen along the lateral cytoplasmic extensions surrounding SCs in DL and PL lobes (Fig. 2Bii, iv, arrows). (Biii,iv) The WGA staining in the DL is less intense (Biii) with lower intensity at cell boundaries (Biv, arrowheads). Asterisk in Biii denotes acellular basement membrane. (Bv) In cross section, the rims of the cup-shaped DL cells make direct contact with the WGA positive duct. (Bvi,vii) Cells of the ML are cup-shaped with the walls of the cup showing low level WGA staining. There is no obvious secretory duct in the ML beyond the proximal tip. Individual channels are denoted “b”–blue, DAPI, “g”- green, tubulin, or “r”–red, WGA. Scale bar lengths are given in microns.

**Figure 3:**
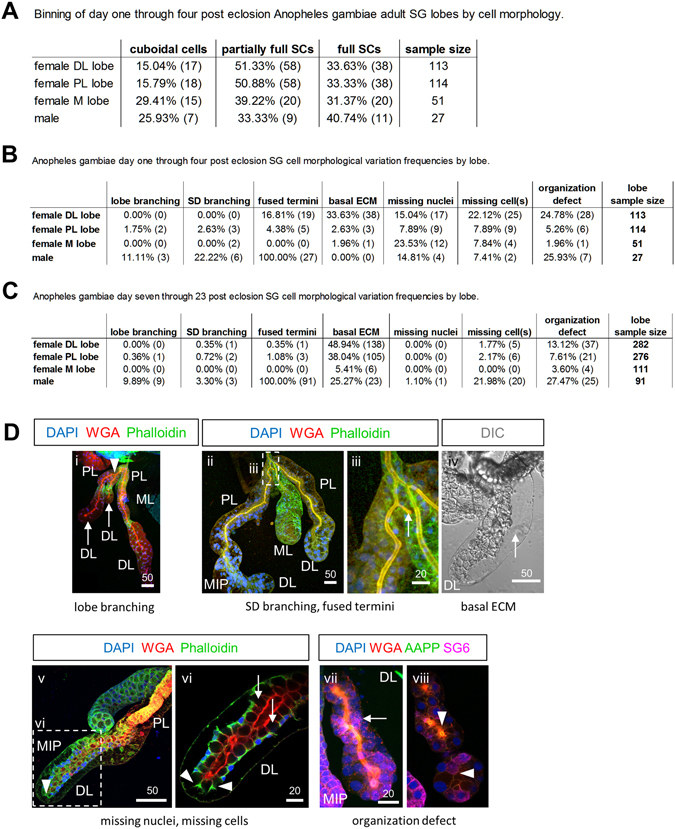
Quantification of *Anopheles gambiae* SG cell and lobe morphologies. (**A**) Binning of SGs by SC phenotype (no SC, partial SC, or full SC) across early adult collections. (**B**) Frequencies of architectural feature variation in early *An. gambiae* salivary glands by lobe. (**C**) Frequencies of architectural feature variation in late *An. gambiae* salivary glands by lobe. (**D**) Representative images of cell morphology phenotypic categories. (i) Lobe branching-branching of an entire salivary gland lobe (duct, lumen, cells, SCs); shown is a proximal bifurcation (arrowhead) of a lateral lobe (arrows). (ii,iii) SD branching, fused terminus-shown is a branched salivary duct without lobe branching (iii) having a fused terminus (iii, arrow). (iv) Basal ECM-acellular space basal to secretory cells (arrow). (v,vi) Missing nuclei, missing cell-the cell body is present, but nucleus is not observed (arrowheads), or entire cells are missing (arrows) from a continuous cell layer surrounding the lumen. (vii,viii) Organization defect-any deviation in tissue organization from the stereotyped single layer of polarized secretory cells surrounding secretory cavities (after PAC fusion) adjacent to a central lumen. Shown is a distal lateral lobe containing disordered multicellular layers (vii, arrow) surrounding a duct where no lumen is present [viii (a central plane), arrowheads]. Antibodies/dyes used are labeled in (**D**). “MIP” images are maximum intensity Z-projections. Scale bar lengths are given in microns.

To learn how SGs mature into previously published morphologies, we next examined adult female SGs 12–36 hours (Fig. 2A) and four days (Fig. 2B) post eclosion (quantified in Fig. 3A). Approximately one day after eclosion, alpha-tubulin levels were more uniform throughout the SG lobes, with notably higher levels in the ML. WGA staining at the SD was high and uniform through most of the lateral lobes and the proximal portion of the ML (Fig. 2Ai). In the PL, secretory cavities (SCs) of various sizes were observed, with cavity size positively correlating with increased basal cell body compression and the straightness of the walls of these cup-shaped cells (Fig. 2Aii). In the DL, SCs were smaller and the basally-positioned cell bodies were less compressed (Fig. 2Ai,iii–v). The morphology of cells in the most distal region of the DL suggested that SCs in some cells had just formed (Fig. 2Aiv). Apical WGA staining was reduced and less uniform in this region, consistent with the mature duct remaining open and not reaching the end of the lateral lobe. In the ML at this stage, small SCs of varying size were visible in the most proximal cells. Cells of the distal ML were slightly flattened and cuboidal with internal PACs, which did not stain for tubulin or WGA (Fig. 2Avi–vi”, arrows). Nuclei were small and positioned at the basal periphery (Fig. 2Avi–vi’). High levels of apical WGA staining were observed in only the more proximal portions of the ML, whereas faint, relatively uniform WGA positive staining was observed in large portions of the ML lumens (Fig. 2Avi,vi’). The morphological variation observed during early SG maturation is further illustrated in Supplementary Videos 1 and 2. Overall, our analysis of SGs approximately one day post eclosion reveal that nearly cells of the lateral lobe are cup-shaped, whereas medial lobe cells mostly remain cuboidal and contain PACs.

By four days post eclosion, *An. gambiae* SG cells in all the lobes had largely achieved the mature cup shaped morphology previously described (Fig. 2Bi). The cup-shaped PL cells surrounded a thick chitinous duct with uniform WGA staining and narrow periductal and inner duct lumena (Fig. 2Bii). Weak WGA signal was observed along the lateral cytoplasmic extensions surrounding SCs in the PL (Fig. 2Bii, arrow). Similarly, most DL cells were cup shaped with compressed basal cell bodies. The ductal WGA staining in the DL was less regular, exhibiting areas with low staining, primarily at cell boundaries (Fig. 2Biii,iv). WGA staining was also observed along the lateral cytoplasmic extensions of each cup shaped cell in the DL (Fig. 2Biv, arrows), except in cells at the very distal end of the tube. The most apical end of the DL cells appeared to directly contact the WGA-positive secretory duct (Fig. 2ABv). As previously noted in *Anopheles stephensi* ^19^, acellular basal regions accompanying compression of the lobe cells were often observed (“Basal ECM”; Figs 2Biii and 3B–D). By this stage, all ML cells were cup shaped with large SCs and a uniform proximal duct that opened into a large shared lumenal space. Cross sections through the distal ML revealed low levels of WGA staining along the walls of these cup-shaped cells (Fig. 2Bvii). Altogether, our findings suggest that female SGs mature in the first few days post eclosion, the lateral lobes mature before the medial lobe, and cells within each lobe mature in a proximal to distal direction.

**Figure 4:**
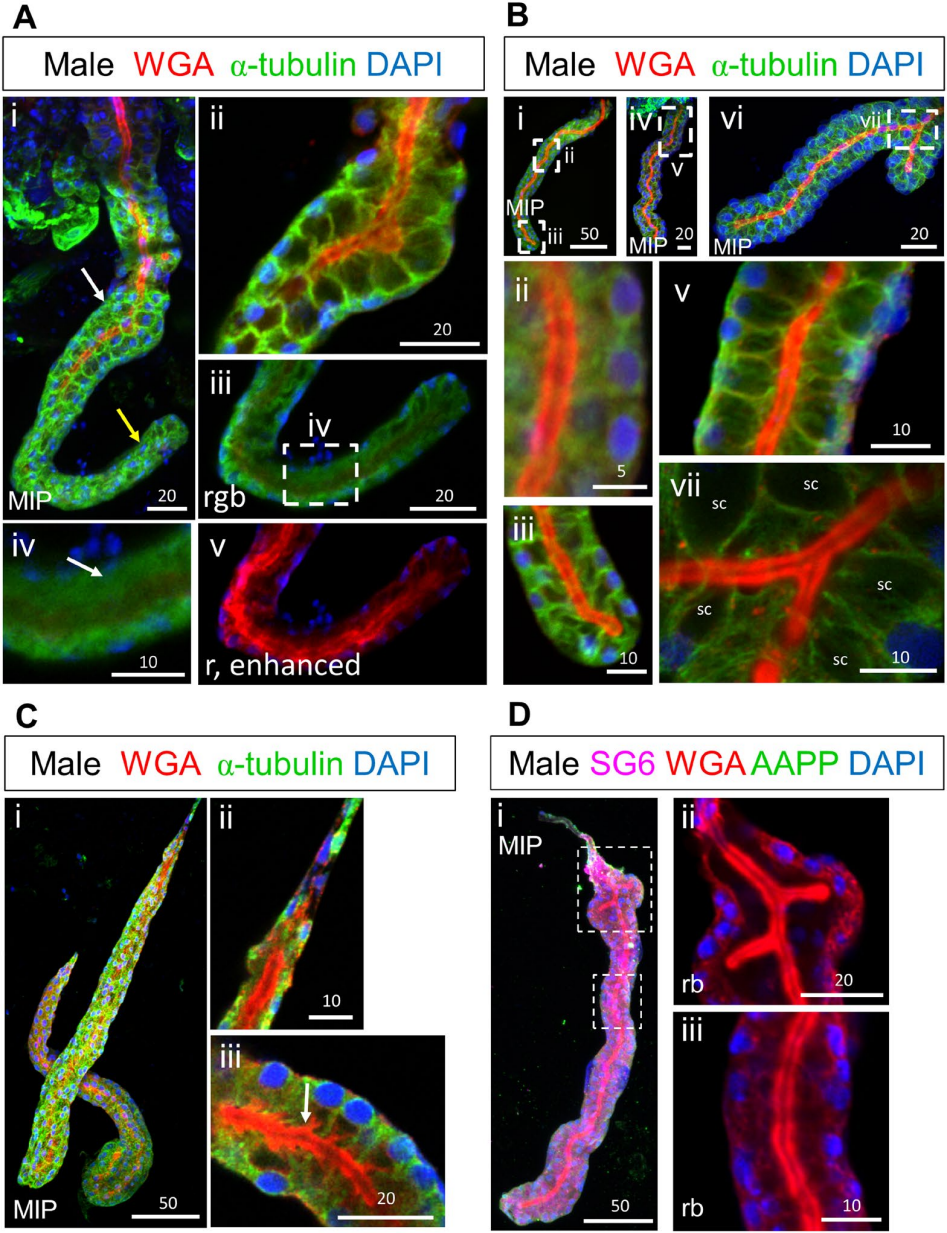
Male adult SG cell maturation mirrors the process in female PLs. Shown are confocal images of male SGs either 30 minutes (**A**), 12–48 hours (**B**), four days (**C**), or 20 days (**D**) post eclosion. In early adult male SGs, SD staining was strongest in the proximal half (Ai, white arrow; Aii) and was nearly absent in the distal half (Ai, yellow arrow; Aiii-v). In regions lacking WGA, cells were largely cuboidal in shape (Aiv, white arrow). With enhanced contrast, WGA staining is variable and discontinuous in the distal region (Av). At 12 hours post eclosion, small SCs are visible throughout the SG (Bi), but the cell bodies are not fully basally compressed (Bii) and with the larger SCs, the surrounding lateral cell extensions are not fully stretched and appear jagged (Biii). At 36 hours post eclosion (Biv), SCs are fully expanded and nuclei/cell bodies are fully basally compressed (Bv). In male SGs, 48 hours post eclosion, with a SD branch (Bvi), cell bodies maintain their organization surrounding SCs, but are positioned in a complex arrangement surrounding the SD on all sides (Bvii). “sc” marks secretory cavities. (**C**) At four days post eclosion (Ci), proximal SD staining was weak (Cii), SCs remained full (Ciii), and evidence of WGA-positive apical release was sometimes observed (Ciii, white arrow). Rarely, older SGs in which SCs were regionally missing were observed (**D**). SD branching is seen in the proximal portion (Dii), WGA signal is robust and continuous throughout (**D**), but proximal cells are cuboidal in shape, while more distal cells surround SCs. SG6 and AAPP staining are observed (Di), suggesting secretion is functional in the proximal region. Individual channels are denoted “p”, “b”, “g”, or “r”. “MIP” images are maximum intensity Z-projections. Scale bar lengths are given in microns.

Male *An. gambiae* SGs appeared to mature along a similar timeline as the female PL, based on images of glands obtained and fixed immediately post eclosion or days later (Figs 3 and 4). Shortly after eclosion, male SG morphology varied considerably along the proximal-distal axis (Fig. 4Ai). Proximal cells tended to have large SCs with basally compressed cell bodies, and robust WGA accumulation along the SD (Fig. 4Aii). In contrast, distal SG cells were largely cuboidal (Fig. 4Aiii,iv, iv, white arrow) with very low levels of irregular lumenal WGA staining at the site of the SD, which was only visible with enhanced contrast (Fig. 4Av). One to two days post eclosion, SG cell shape was more consistent, with cup-shaped cells throughout the length of the tube (Fig. 4B). Some SG cells had small SCs and little or no basal compression (Fig. 4Bii), whereas others had larger SCs that were not quite full, as evidenced by the jagged lateral extensions (Fig. 4Biii). Nonetheless, by this stage, SD WGA staining was robust throughout the length of the SG in all samples. SCs from male SGs had fully expanded by day two post eclosion (Fig. 4Biv,v). SG morphology did not change substantially after day two (Fig. 4C), but apical accumulations of WGA-positive secretions were seen at day four (Fig. 4Ciii). Rarely, older male SGs had regional instances of cuboidal cells surrounding an open lumen (Figs 3C and 4D). Unlike in *An. stephensi* ^19^, where extensive branching of male SGs was observed, only about 10% of male glands showed lobe branching, with about twice as many showing duct branching (Fig. 4B,C). All salivary ducts were closed in male SGs, regardless of whether the duct was branched or not. In contrast, ~83% of ducts were open in females. Other variations in SG architecture were also observed in both male and female SGs, indicating a similar level of morphological variation as observed with *An. stephensi* (Fig. 3B,C)^19^.

### Pre-apical compartments fuse with the apical surface to form secretory cavities

**Figure 5:**
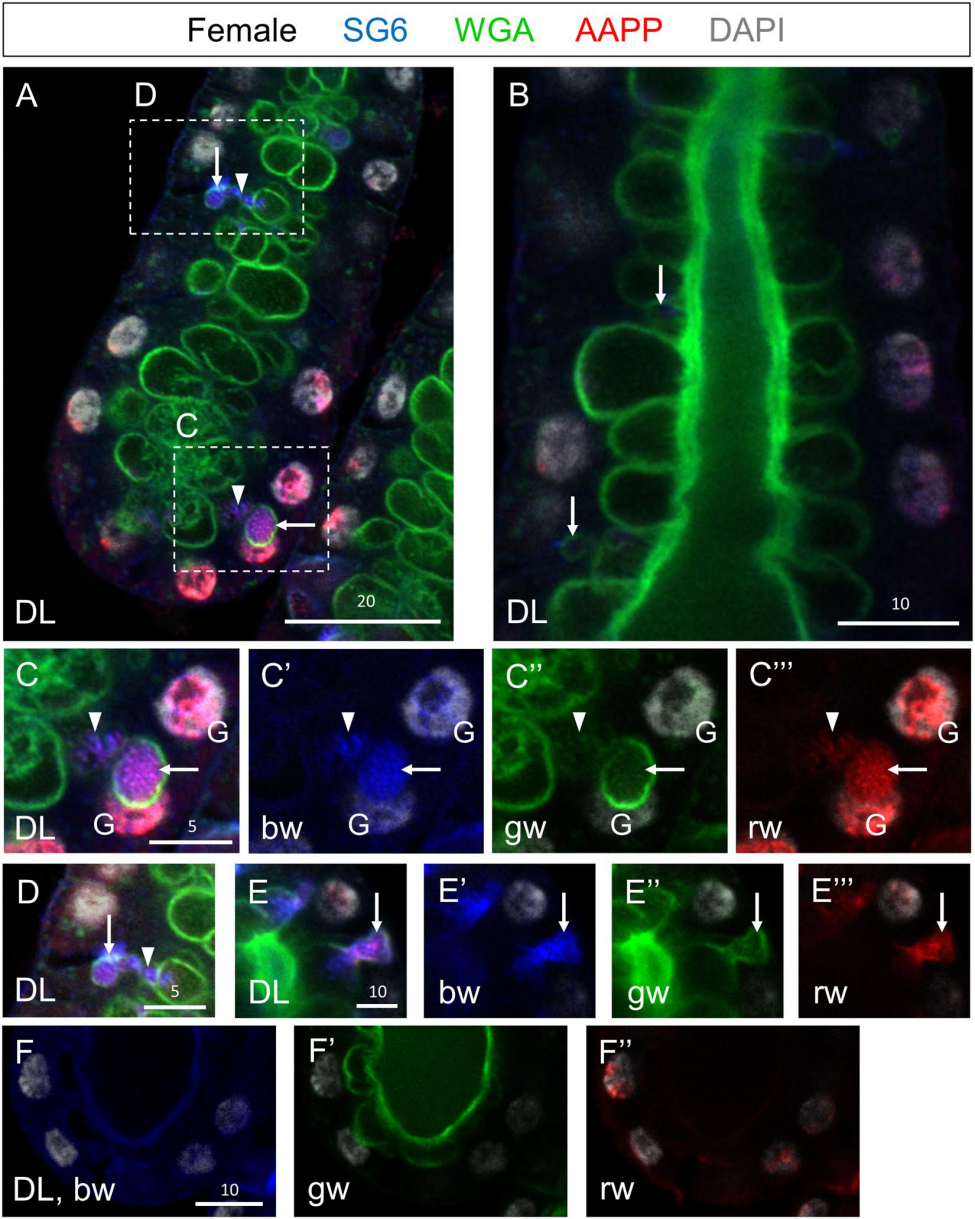
Early adult SG DL stained with antibodies to the secreted proteins SG6 (blue) and AAPP (red), and stained with the dyes WGA (green–O-GlcNAcylated proteins and chitin) and DAPI (white, DNA). (**A**) High magnification confocal slice of the DL at a plane between the apical and basal surface reveals large secretory cavities lined with WGA staining as well as occasional PACs (arrows). (**B**) High magnification confocal slice of the DL at a plane of the apical surface reveals large secretory cavities lined with WGA staining as well as occasional PACs (arrows). (**C**) A WGA-, SG6-, and AAPP-positive PAC is observed adjacent to the nucleus (arrow). Small vesicle-like buds are seen between the PAC and the apical surface/SC (**A**,**C**,**D**, arrowheads). (**D**) A smaller PAC with more vesicles between the PAC and the secretory cavity. (**E**) A PAC (arrow) is observed fusing to a secretory cavity. Individual channels are denoted “p”, “b”, “g”, or “r”. “G” indicates perinuclear staining that is likely to correspond to the Golgi. Scale bar lengths are given in microns.

**Figure 6:**
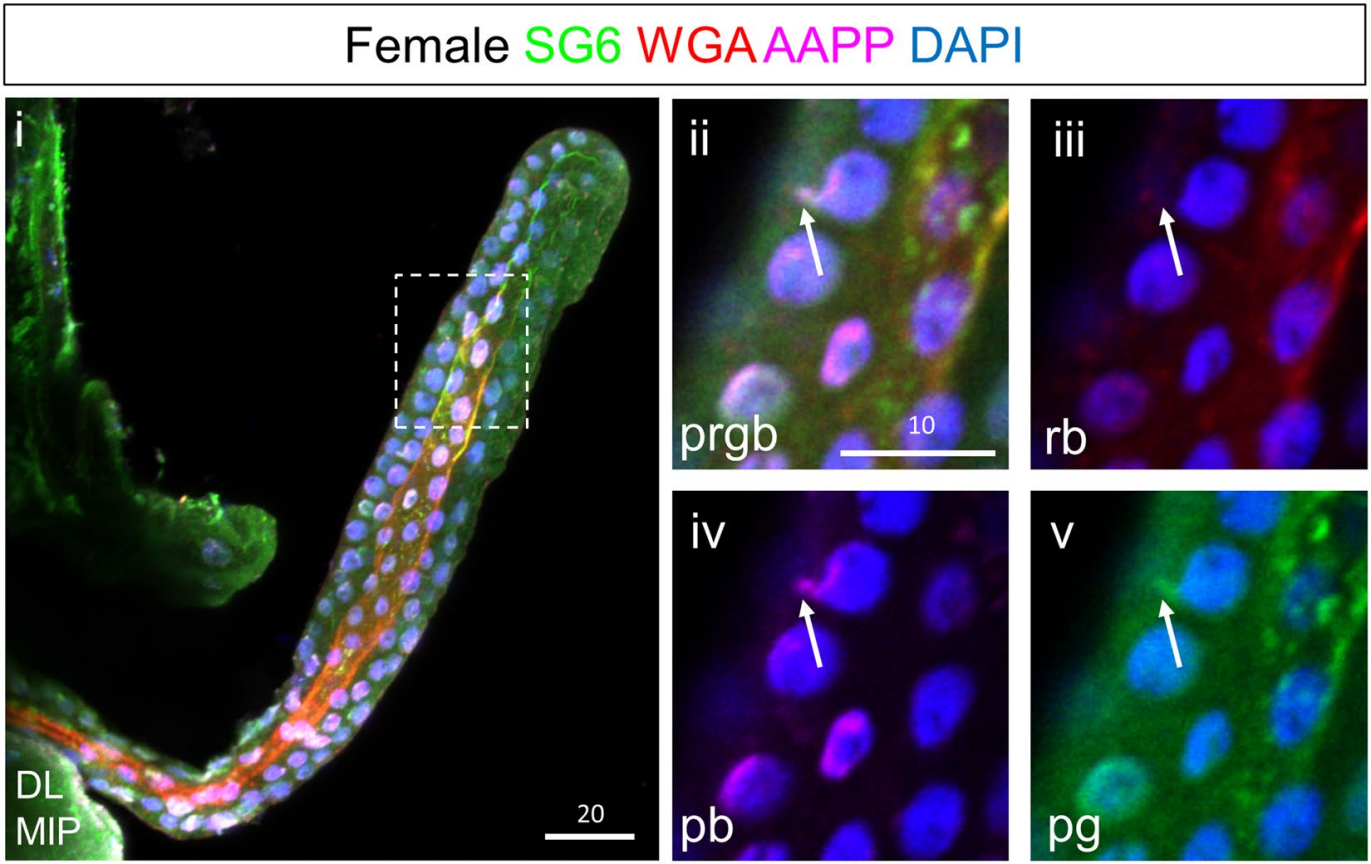
Small pre-apical compartments are located near the nucleus and presumptive Golgi prior to expansion. Shown are confocal images from a female SG DL lobe around 12 hours post eclosion. Apical WGA staining is weak through most of the DL lobe (i). Some nuclei show small accumulations of secretory proteins and weak WGA in the absence of DNA, near the nucleus and presumptive Golgi (ii–v, arrow). These may be sites of pre-apical compartment formation. Individual channels are denoted “p”, “b”, “g”, or “r”. “MIP” images are maximum intensity Z-projections. Scale bar lengths are given in microns.

To gain insight into how SG cells transition from cuboidal to cup shaped, we examined DLs from adults within 24 hours post eclosion stained with WGA and two secreted salivary proteins, SG6^20^ and AAPP^21^. In DLs with newly formed SCs, prominent WGA staining was observed lining the SCs and was also sometimes observed in two parallel rows along the central duct (Fig. 5A,B; green channel). WGA staining was also observed on the periphery of PACs, which had a range of morphologies at this stage (Fig. 5C–E). Low level WGA staining was detected in vesicular structures between the PACs and the apical surfaces (Fig. 5A–D, arrowheads). AAPP staining was most prominent in perinuclear structures in the DLs, which, based on morphology, are likely to be Golgi (Figs 5A,B and 6 and Supplementary Video S3). Low-level vesicular staining of AAPP was also observed basally in the cell bodies, where similar staining with the SG6 antiserum was observed (Supplementary Video S3, most basal focal plane). High levels of both AAPP and SG6 were observed in the WGA-lined PACs (Fig. 5, arrows) and in vesicular structures between the PACs and apical cell surface (Fig. 5A–D, arrowheads). Altogether, these data suggest that PACs are large secretory compartments functionally equivalent to an apical lumen. PACs contain apically-secreted proteins, and PACs are almost always lined with apical chitin.

**Figure 7:**
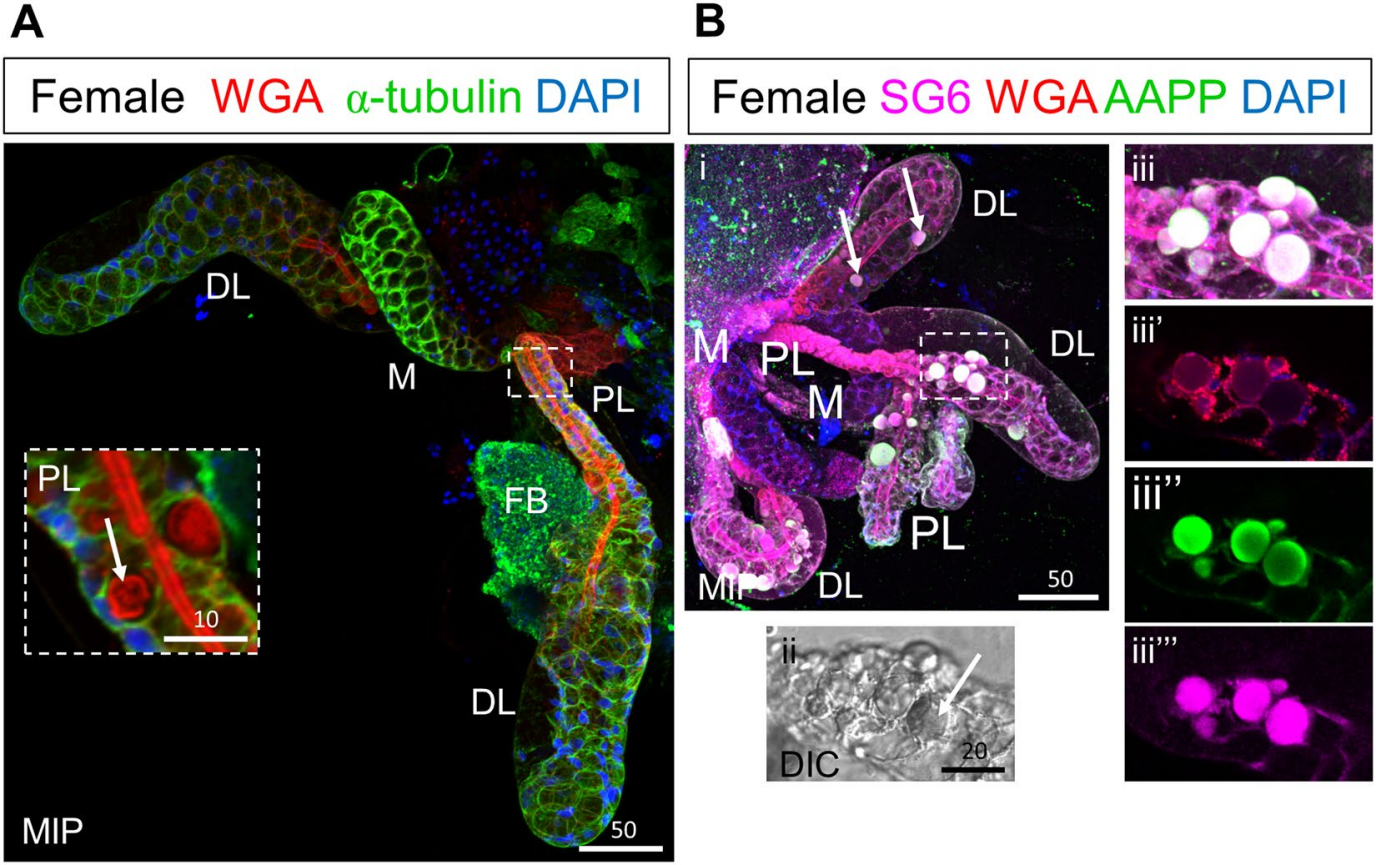
Secretory bodies are also observed in later adulthood. Shown are confocal images of female SGs 14 (**A**), or 20 (**B**) days post eclosion stained with the dyes WGA and DAPI and antibodies against either alpha-tubulin (**A**) or the saliva proteins SG6 and AAPP (**B**). At these late time points, large WGA-positive secretory bodies are observed (**A**,**B**, arrows), sometimes within the SC (**A**, inset, arrow). WGA enrichment and secretory body number varied greatly with age and among individual SGs. Both SG6 and AAPP are present in secretory bodies (Biii,vi), but newly synthesized alpha-tubulin was largely excluded (**A**, inset). “FB” refers to fat body tissue. Individual channels are denoted “p”, “b”, “g”, or “r”. “MIP” images are maximum intensity Z-projections. Scale bar lengths are given in microns.

What are likely to be early stage PACs were seen in SGs isolated from a subset of early female SGs (approximately 12 h post eclosion). Small WGA-AAPP-SG6-positive structures were observed in close contact with the AAPP-positive Golgi staining associated with the nucleus (Fig. 6). PAC-like structures were also observed in the SGs of late stage sugar fed males and females SG, at 14 (Fig. 7A), and 20 days (Fig. 7B) post eclosion. These large compartments containing SG6, AAPP, and variable levels of WGA were most often found in the DLs, appearing as phase dark compared with the surrounding SG tissue (Fig. 7Bii, arrow). Late stage PACs could be seen singularly (Fig. 7Bi, arrows) or in clusters (Fig. 7).

**Figure 8:**
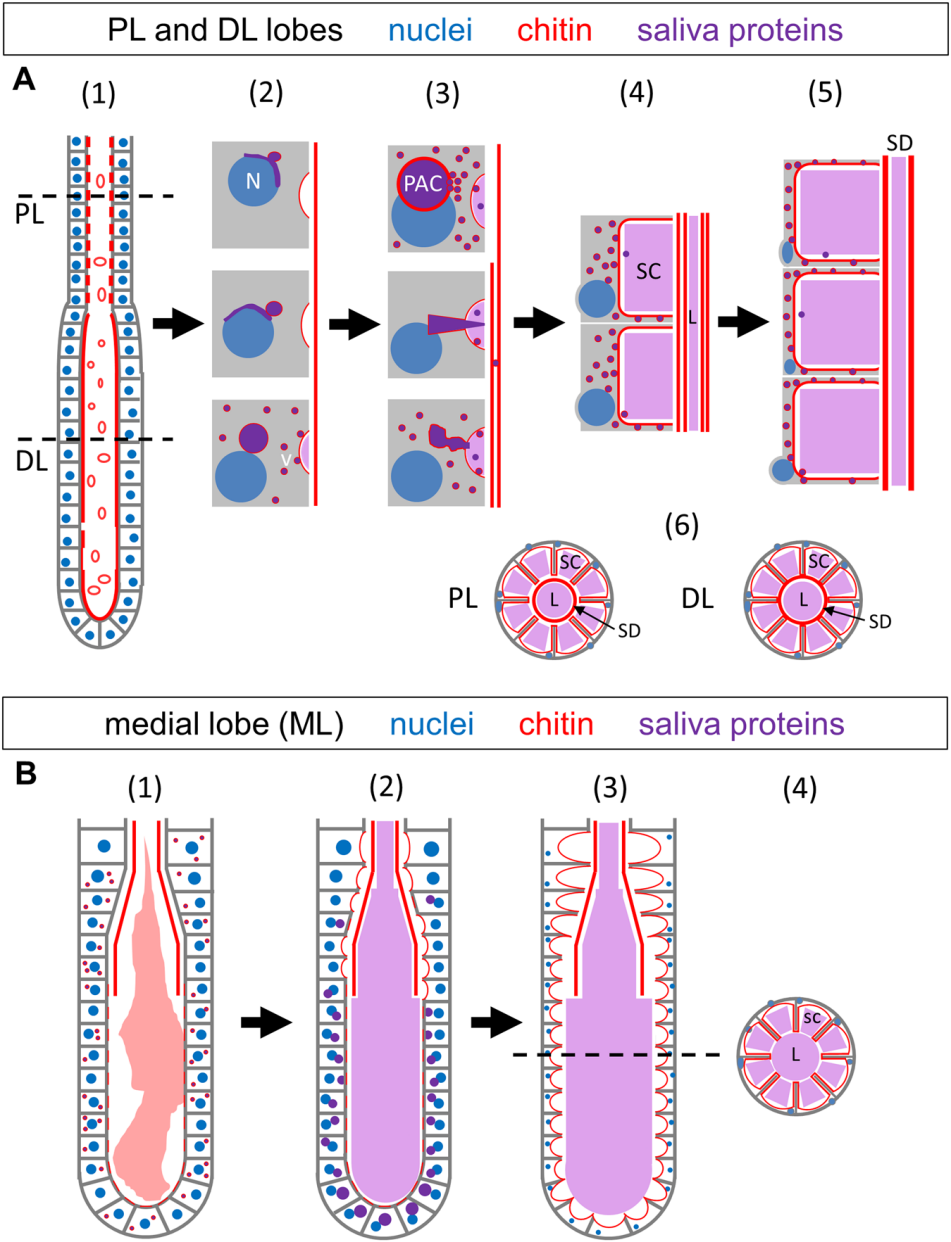
Model of salivary gland cellular maturation and duct formation. (**A**) Lateral lobe maturation. SG secretory cells in newly eclosed adults are nearly cuboidal in shape (A1,B1). Within the first 36 hours post eclosion, a pre-apical compartment (PAC) positive for WGA and secreted proteins forms; vesicles (v) positive for WGA and saliva proteins are also observed (2). Buds of secreted material are observed between the PAC and the growing apical surface/SC (3). PAC fusion to the apical plasma membrane is observed, releasing remaining secretory proteins (3). PAC fusion expands the apical surface of the cell and the SC (4). Continued secretion ultimately leads to massive SC expansion and basal compression of the remaining cellular material (5). By day four, a small periductal space separates the walls of the cup shaped cells from the central duct in the PL but not DL (6). Secretion-positive vesicles are rarely observed intact in the SC and SD, perhaps due to denaturation and/or dilution in the presumptive acidic lumenal compartment. (**B**) Medial lobe maturation. In the earliest adult SG medial lobes, a fibrous matrix can be observed within part of the lumenal space, and sites of interaction are observed at the apical surface of cells (1). The matrix is continually shrinking in size and loses cell contact, until the matrix is no longer visible and PACs begin to form and fuse to the apical surface (2). At day four post eclosion and beyond, SCs have formed and expanded throughout the SG (3). In cross-section (4), cup shaped cells surround secretory cavities open to the lumen. Lumenal saliva is marked in light purple. Abbreviations: N–nucleus; SC–secretory cavity; PAC–pre-apical compartment; SD–salivary duct; L–lumen.

Based on the staining patterns of WGA and the secretory markers AAPP and SG6 at multiple stages, as well as the variety of PAC structures in the process of fusing with the apical surface, we propose a model in which either direct fusion of PACs or indirect vesicle-mediated fusion of PACs to the apical surfaces converts the cuboidal cells of the immature adult SG into the cup-shaped functional cells observed in the mature structure (Fig. 8). Chitin synthesis is known to occur at the apical plasma membrane, where the activated precursor (UDP-GlcNAc) is converted by an integral membrane protein [Chitin Synthase (CHS)] into chitin fibrils^22^. Upon synthesis, the chitin fibrils are translocated across the plasma membrane and deposited on extracellular surfaces. Finding high levels of WGA staining on the apical surfaces of the immature cuboidal SG cells, lining the surfaces of PACs, and lining the surfaces of newly formed SCs argues that PACs are a “pre-apical compartment” equivalent to the apical lumen. The high concentrations of the two apically-secreted proteins, AAPP and SG6, in PACs further supports this model. PAC fusion with the existing apical surface also explains the double row of chitin staining sometimes observed near the maturing secretory duct in early DLs.

The PACs described herein are likely related to the subapical compartments (SAC) that have been described during the maturation of mammalian hepatocytes and in both MDCK and HUVEC cells as they organize an apical lumen^23^. Hoekstra *et al*. hypothesized that SACs contain specific complements of proteins and lipids that provide an intracellular membrane pool that can be readily recruited for the assembly of specialized plasma membrane domains^23^. In the case of mosquito SGs, the PACs serve the purpose of rapidly converting the more simple early cuboidal morphology of SG cells into the cup-shaped mature and functional morphology. PACs provide both a bolus of secretory content and a huge supply of already polarized membrane to surround the SC. The large quantities of enzymes and other molecules stored in SCs can thus be immediately accessed to facilitate blood feeding. The presence of these structures in late stage adult mosquitoes could indicate that PAC formation and fusion is an ongoing process, even in sugar fed animals, and might occur as new cells mature to replace dead or dying cells.

These studies also provide insight into how the secretory duct (SD) is formed. Two hypotheses could have explained formation of this structure. First, the SD could arise from a cytoplasmic extension originating in TDJ duct cells and extending through the SG, hollowing, and serving as a template for chitin deposition. This mechanism would be akin to that proposed for lumen formation in the developing *Drosophila* tracheoles^24^. Alternatively, secretory cells could form the SD, a possibility that was difficult to reconcile with the unusual cup-shaped morphology of the mature cells. Our discovery that secretory cells begin as cuboidal structures that secrete chitin directly from their apical surfaces indicates that these cells are the source of the developing duct. The immature duct directly contacts the secretory cell surface (Figs 1C and 4A), and only separates from that surface as the PACs fuse to form secretory cavities. Subsequent additional chitin secretion then adds to the already templated SD. Thus, our findings reveal how the functional morphology of mature secretory cells is achieved as well as providing insight into secretory duct assembly. The processes of cell shape change and salivary duct maturation may ready the mosquito for robust feeding, while simultaneously providing a depot for the accumulation of infective pathogens. Uncovering the genetic circuits responsible for adult SG maturation should allow for novel strategies to block pathogen transmission.

## Methods

### Mosquitoes

*Anopheles gambiae* (Keele) mosquitoes were obtained from the Johns Hopkins University Bloomberg School of Public Health Malaria Research Institute Insectary as pupae and maintained on 10% sucrose *ad libitum* following eclosion.

### Antibodies and dyes

Antibodies and dyes employed in this study included: anti-alpha-tubulin (1:10, AA4.3, DSHB), anti-gSG6 (1:100, gift from Hiroyuki Matsuoka), anti-AAPP (1:50, gift from Fabrizio Lombardo), WGA (1:40, Vector Laboratories), DAPI (1:60, Life Technologies).

### Immunohistochemistry

Salivary gland immunohistochemistry was performed as in our previous study in *Anopheles stephensi* ^19^, with the following modifications. Briefly, SGs attached to heads were dissected into 1X PBS from adult mosquitoes either: 30 minutes post eclosion, 12 hours post eclosion, 24 hours post eclosion, 36 hours post eclosion, four days post eclosion, seven days post eclosion, 10 days post eclosion, 14 days post eclosion, or 20 days post eclosion and placed on ice. Following dissections, PBS was removed and SGs were washed with cold 100% acetone for 75 seconds, and then washed with 1X PBS. Samples were incubated with primary antibody in 1X PBS at 4 degrees C overnight, then washed 2X with 1X PBS. Secondary antibody incubation was at room temperature for two hours. SGs were counterstained with DAPI and WGA half an hour prior to the end of secondary antibody incubation. SGs were then gently rinsed in 1X PBS two times prior to mounting in 100% glycerol on SuperFrost+ slides (VWR). Slides were stored at 4 degrees C prior to imaging.

### Confocal Microscopy

Imaging was conducted using either a Zeiss LSM700 or LSM780 laser scanning confocal microscope housed in the Johns Hopkins University School of Medicine Microscope Facility. The step size used in 3D image stack captures was one micron. Each figure is composed of representative images from between six and 20 imaged glands, selected from 30 to 200 dissected and stained glands per experiment, processed between one and four different days. Image processing was completed in Zeiss Zen 2010 and Adobe Photoshop CS4.

## Electronic supplementary material

Supplemental Information

Supplemental Video 1

Supplemental Video 2

Supplemental Video 3
