# Supplementary material for: Salivary gland maturation and duct formation in the African malaria mosquito Anopheles gambiae
Source: Sci Rep. 2017 Apr 4;7:601. doi: 10.1038/s41598-017-00672-0 (PMC5428862; doi:10.1038/s41598-017-00672-0)
Supplement: Supplementary file 1 — Supplemental Information [file 41598_2017_672_MOESM1_ESM.pdf]

Salivary gland maturation and duct formation in the African malaria mosquito *Anopheles gambiae*

Michael B. Wells, Jordan Villamor, and Deborah J. Andrew[1]

[1] corresponding author
The Johns Hopkins University School of Medicine
Department of Cell Biology
725 N. Wolfe St.
Baltimore, MD 21205

Phone: 410-614-2722
Fax: 410-955-4129
email: dandrew@jhmi.edu

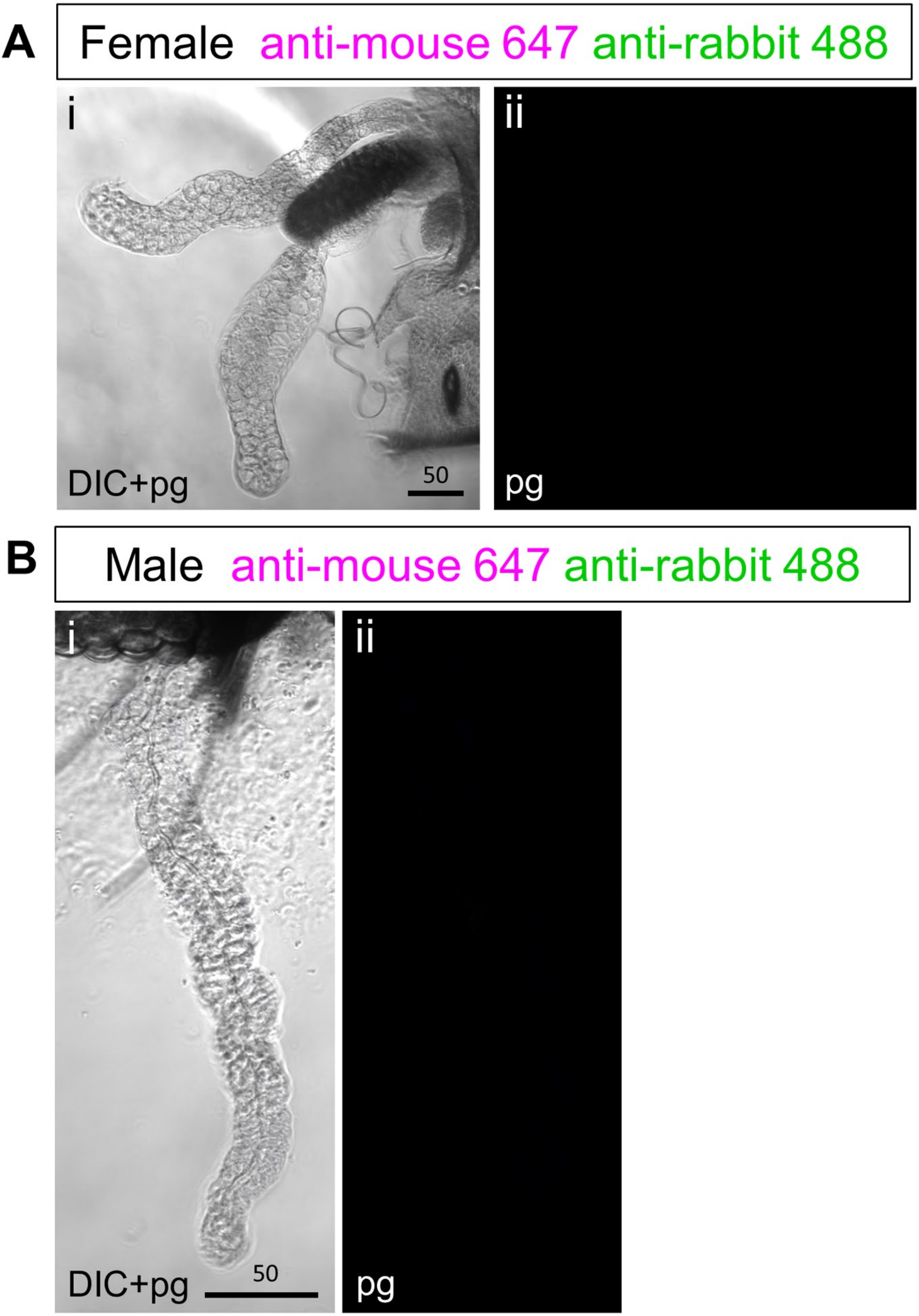

A
Female anti-mouse 647 anti-rabbit 488
i
ii
DIC+pg
50
pg
B
Male anti-mouse 647 anti-rabbit 488
i
ii
DIC+pg
50
pg

Supplementary Figure S1. No primary antibody controls for immunostaining. Shown are confocal images of either female (A) or male (B) adult SGs 10 days post eclosion stained with only secondary antibodies at the concentrations used for all experiments (anti-rabbit Alexa 488 and anti-mouse Alexa 647). Images were taken using the same microscope settings as in experiments that included primary antibodies and were imaged in parallel (Ai-ii, Bi-ii). Scale bar lengths are given in microns.

Supplementary Video S1. DL cell shape Z-stack example 1.

Supplementary Video S2. DL cell shape/dead or dividing cell Z-stack example 2.

Supplementary Video S3. DL secretory marker / PAC, SC shape example Z-stack.
